# Primary left ventricular intimal sarcoma: a case report of multimodality imaging and molecular confirmation

**DOI:** 10.3389/fcvm.2026.1964010

**Published:** 2026-09-30

**Authors:** Peiyan Peng, Liangyu Wang, Bingli Li, Peixiu Yao, Zexin Zhang

**Affiliations:** 1 Department of Ultrasound, Shantou Central Hospital, Shantou, Guangdong, China; 2 Department of Radiology, Shantou Central Hospital, Shantou, Guangdong, China; 3 Department of Pathology, Shantou Central Hospital, Shantou, Guangdong, China; 4 Department of Emergency Medicine, Shantou Central Hospital, Shantou, Guangdong, China

**Keywords:** cardiac computed tomography angiography, cardiac intimal sarcoma, CDK4 amplification, left ventricular mass, MDM2 amplification, transesophageal echocardiography, transthoracic echocardiography

## Abstract

Primary cardiac intimal sarcoma is a rare malignant mesenchymal neoplasm whose intracavitary appearance may mimic other cardiac masses. We report a woman in her early 60s with recurrent palpitations and occasional exertional dyspnea. Transthoracic echocardiography (TTE) demonstrated a giant, highly mobile, irregular multilobulated mass occupying most of the left ventricular cavity, with slit-like interlobular clefts and a probable broad-based attachment near the apical interventricular septum. Low-velocity color Doppler demonstrated residual flow around and between lobules, while continuous-wave Doppler showed a peak left ventricular outflow tract velocity of 3.09 m/s and a peak instantaneous gradient of 38 mmHg. Cardiac computed tomography angiography (CTA) showed a large relatively hypoattenuating left ventricular mass with heterogeneous enhancement: some portions showed no obvious enhancement, whereas others showed mild enhancement; the interface with the ventricular wall was locally indistinct, so myocardial involvement could not be excluded. After induction of general anesthesia, intraoperative transesophageal echocardiography (TEE) showed the mass nearly filling the left ventricular cavity, but the attachment and fine multilobulated architecture were less clearly seen than on preoperative TTE. The patient underwent tumor resection with concomitant mitral valve repair. Histology showed mildly atypical spindle cells with fascicular and focal perivascular growth, readily identified mitotic figures, and mild myxoid stromal change. Immunohistochemistry showed MDM2 positivity, and fluorescence *in situ* hybridization demonstrated MDM2 and CDK4 amplification, supporting the diagnosis of intimal sarcoma. At approximately 2 weeks after surgery, follow-up TTE showed no residual or recurrent left ventricular mass, preserved left ventricular systolic function, minimal residual mitral regurgitation, and a peak outflow tract velocity of 0.92 m/s without residual obstruction. Postoperative 18F-FDG PET/CT performed at approximately the same time identified no definite extracardiac primary tumor or obvious distant metastatic lesions. The case illustrates how multimodality imaging can define the anatomy and hemodynamic effects of a rare left ventricular intimal sarcoma, while histopathologic and molecular findings remain essential for definitive diagnosis.

## Introduction

Primary cardiac intimal sarcoma is a rare and aggressive malignant mesenchymal neoplasm with nonspecific clinical and imaging manifestations. Intracavitary lesions may be mistaken for myxoma, thrombus, or other primary cardiac tumors. Although multimodality imaging is important for defining anatomic relationships and planning intervention, definitive diagnosis generally requires integration of histomorphology, immunohistochemistry, and molecular findings. Amplification of MDM2, often accompanied by co-amplification of cell-cycle genes such as CDK4, is an important diagnostic feature (1–3).

We report a giant primary left ventricular intimal sarcoma with a complex multilobulated appearance. TTE, TEE, and cardiac CTA each contributed different information about tumor morphology, attachment, and hemodynamic effects. Definitive diagnosis was based on histopathologic, immunohistochemical, and molecular findings. The difference between preoperative TTE and intraoperative TEE in this patient should be interpreted in light of the different imaging conditions and physiologic states.

## Case description

A woman in her early early 60s was admitted with a 1-month history of recurrent palpitations. Episodes lasted from several minutes to several hours and resolved spontaneously with rest. She also reported occasional exertional dyspnea but denied chest pain, syncope, hemoptysis, or paroxysmal nocturnal dyspnea. Her medical history was notable for type 2 diabetes mellitus treated with oral hypoglycemic agents, without known hypertension or coronary artery disease. On examination, she was alert, with a regular cardiac rhythm, no audible pulmonary rales, and no lower-extremity edema.

Electrocardiography showed sinus rhythm, increased left ventricular voltage, and ST-T abnormalities. Laboratory testing showed hemoglobin 117 g/L, high-sensitivity C-reactive protein 6.04 mg/L, cardiac troponin I 0.161 ng/mL, and B-type natriuretic peptide 39.40 pg/mL. White blood cell and platelet counts, serum electrolytes, and hepatic and renal function were unremarkable. The clinical course and key diagnostic and therapeutic events are summarized in Table 1.

**Table 1:** Timeline.

Time point	Clinical event	Key findings/management
∼1 month before admission	Symptom onset	Recurrent palpitations; occasional exertional dyspnea.
Admission	Initial clinical assessment	Sinus rhythm with ST-T abnormalities; mild laboratory abnormalities as described in the text.
Preoperative assessment	TTE	Giant multilobulated left ventricular mass; severe mitral regurgitation; LVOT peak velocity 3.09 m/s and peak gradient 38 mmHg.
Preoperative assessment	Cardiac CTA	Large left ventricular mass related to the apex and apical interventricular septum; heterogeneous enhancement with nonenhancing and mildly enhancing portions; locally indistinct interface with the ventricular wall, so focal myocardial involvement could not be excluded; no definite extracardiac extension.
After induction of anesthesia	Intraoperative TEE	Mass nearly occupied the entire left ventricular cavity; the attachment site and detailed multilobulated architecture were less clearly visualized than on preoperative TTE.
Surgery	Tumor resection and mitral repair	Thoracoscopic-assisted resection under cardiopulmonary bypass; 28-mm prosthetic annuloplasty ring.
Post-resection	TEE	No visible residual left ventricular mass.
Pathologic assessment	Histology, immunohistochemistry, and FISH	Mildly atypical spindle cells with fascicular and focal perivascular growth, readily identified mitotic figures, mild myxoid stromal change, and no tumor necrosis; MDM2 immunopositivity with MDM2 and CDK4 amplification supported intimal sarcoma.
∼2 weeks after surgery	Follow-up TTE	No residual/recurrent mass; preserved LV systolic function; minimal MR; LVOT peak velocity 0.92 m/s with no residual obstruction; clinically stable during early postoperative assessment.
∼2 weeks after surgery	18F-FDG PET/CT systemic staging	No definite extracardiac primary tumor or obvious distant metastatic lesions; no abnormal hypermetabolic activity suggestive of residual tumor in the left ventricular surgical region.
Postoperative period	Postoperative multidisciplinary review	Microscopic residual tumor at the surgical margin could not be excluded. Postoperative systemic therapy was recommended, with chemotherapy planned to begin approximately 3 weeks after surgery; serial chest and upper abdominal CT was recommended after every two treatment cycles.

### Diagnostic assessment

Preoperative TTE demonstrated a giant heterogeneous mass occupying most of the left ventricular cavity. Left ventricular systolic function was preserved, with an ejection fraction of 74%, and severe mitral regurgitation was present. On representative two-dimensional images, the mass measured approximately 58.6 mm × 34.0 mm. It was irregular, highly mobile, and composed of multiple lobules separated by distinct slit-like clefts. Real-time imaging suggested a broad-based attachment near the apical interventricular septum, which was more readily appreciated dynamically than on any single still image. Two-dimensional pseudocolor imaging helped delineate the lobular contours and clefts, while real-time three-dimensional TTE provided complementary volumetric information (Figure 1; Supplementary Videos S1 and S2).

**Figure 1:**
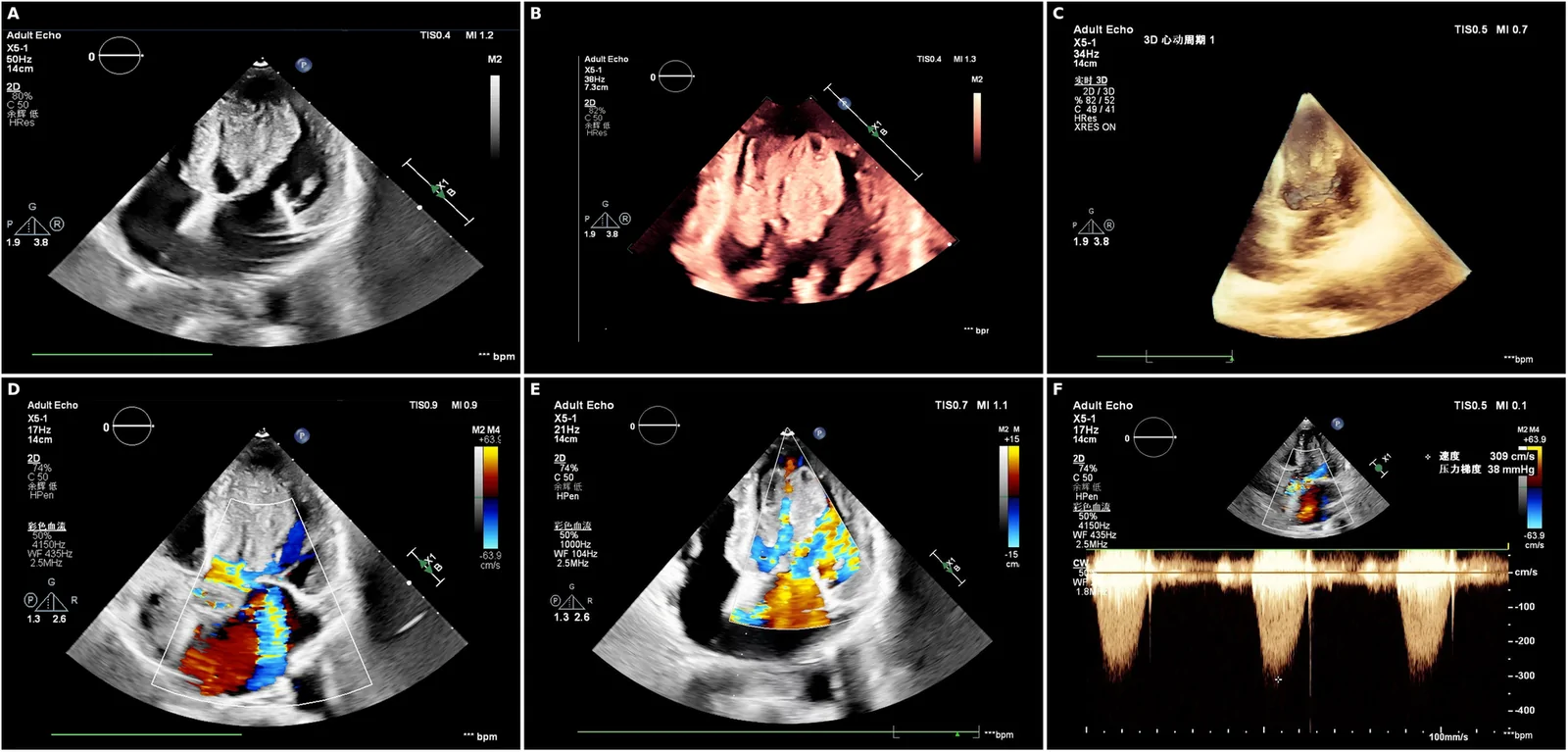
Multimodal transthoracic echocardiographic features of the left ventricular mass. **(A)** Conventional two-dimensional grayscale echocardiography shows a giant mass occupying most of the left ventricular cavity. **(B)** Two-dimensional pseudocolor imaging highlights the irregular multilobulated contour and slit-like spaces between adjacent lobules. **(C)** Real-time three-dimensional echocardiography demonstrates the volumetric configuration and intracavitary extent of the mass. **(D)** Color Doppler imaging demonstrates flow acceleration at the obstructed left ventricular outflow tract. **(E)** Low-velocity-scale color Doppler demonstrates residual low-velocity blood flow within some interlobular clefts. **(F)** Continuous-wave Doppler demonstrates a peak left ventricular outflow tract velocity of 3.09 m/s and a peak instantaneous pressure gradient of 38 mmHg.

Color Doppler imaging demonstrated the relationship between the mass and the left ventricular outflow tract, with flow acceleration at the obstructed region. At a low color Doppler velocity scale, residual low-velocity blood flow was visible within some interlobular clefts (Figure 1; Supplementary Video S3). Continuous-wave Doppler across the left ventricular outflow tract demonstrated a peak velocity of 3.09 m/s and a peak instantaneous pressure gradient of 38 mmHg, consistent with mild left ventricular outflow tract obstruction caused by the mass.

Cardiac CTA demonstrated an irregular, relatively hypoattenuating left ventricular mass measuring approximately 59 mm × 31 mm × 32 mm. The lesion extended along the long axis of the left ventricle and was closely related to the apex and apical interventricular septum. Enhancement was heterogeneous: some portions showed no obvious enhancement, whereas other portions showed mild enhancement. The interface with the adjacent ventricular wall was locally indistinct; therefore, focal myocardial involvement could not be excluded on CTA. No definite extracardiac extension was identified, and no significant obstructive coronary artery disease was seen (Figure 2). Cardiac magnetic resonance (CMR) was not performed preoperatively because the clinical team considered that the large, highly mobile left ventricular mass posed a potential hemodynamic risk and warranted prompt surgical intervention. The available TTE and cardiac CT/CTA findings provided sufficient information on tumor size, location, mobility, and its relationship with adjacent cardiac structures for immediate operative planning; therefore, additional CMR was unlikely to alter the immediate management strategy.

**Figure 2:**
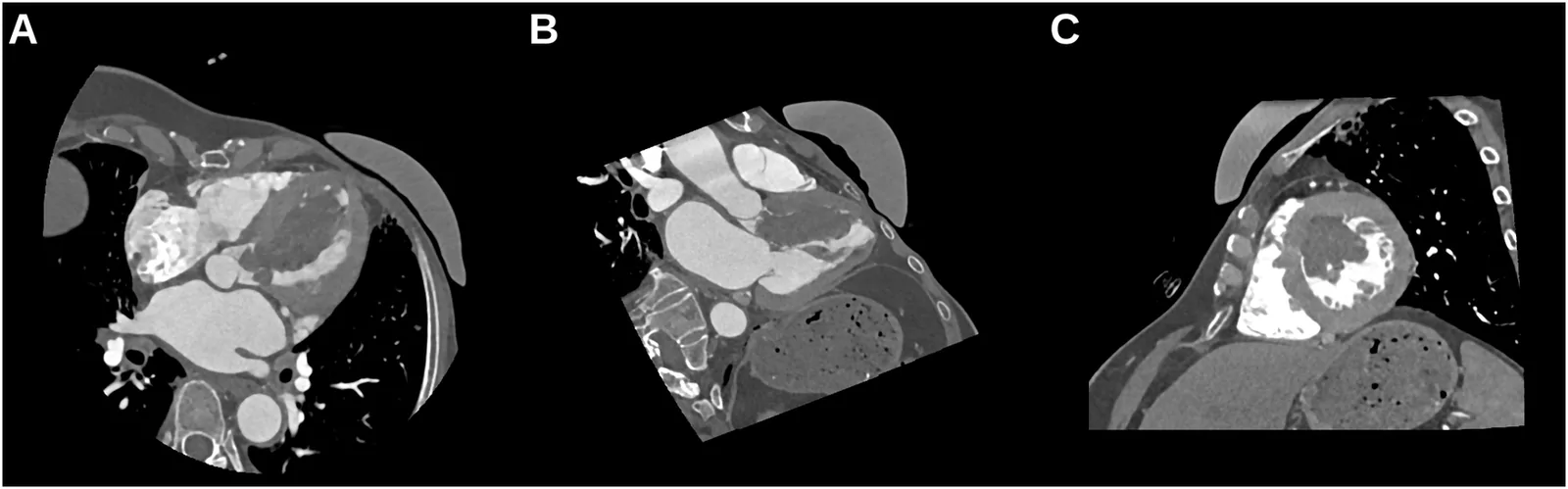
Cardiac computed tomographic angiographic findings. **(A)** Four-chamber view demonstrates the overall extent of the large left ventricular mass and its relationship to the apex and interventricular septum. **(B)** Three-chamber/left ventricular long-axis reconstruction demonstrates extension of the mass along the left ventricular long axis and its spatial relationship to the left ventricular outflow tract. **(C)** Short-axis view demonstrates the large intracavitary tumor burden and the interface between the mass and adjacent ventricular myocardium. The lesion was relatively hypoattenuating and showed heterogeneous enhancement, with nonenhancing and mildly enhancing portions. The interface with the ventricular wall was locally indistinct, so focal myocardial involvement could not be excluded; no definite extracardiac extension was identified.

After induction of general anesthesia, pre-resection intraoperative biplane TEE showed the tumor nearly filling the left ventricular cavity (Supplementary Video S4). The attachment and detailed multilobulated configuration were less clearly seen than on preoperative TTE. Several factors may have contributed, including differences in acoustic windows, imaging planes, and probe orientation. The apical location of the attachment also placed the relevant left ventricular structures in the far field of the transesophageal acoustic window. In addition, anesthesia-related changes in ventricular loading and chamber geometry may have altered the relation between the mass and the ventricular wall. Real-time 3D TEE was not performed in this case.

The preoperative differential diagnosis included myxoma, thrombus, papillary fibroelastoma, and other primary or metastatic cardiac tumors. Myxomas are typically atrial in location and often arise from a relatively discrete attachment, whereas papillary fibroelastomas are usually small, highly mobile, and valvular in origin. Thrombi may occur in the left ventricle, particularly in the setting of regional wall-motion abnormalities or ventricular dysfunction, but the preserved left ventricular systolic function, marked mobility, complex multilobulated morphology, and broad-based apical attachment in this case were less typical of thrombus. Intimal sarcoma may present as a large intracavitary or infiltrative mass with heterogeneous morphology and a broad attachment. In the present case, the combination of large tumor burden, multilobulated architecture with interlobular clefts, broad-based apical attachment, and locally indistinct myocardial interface raised concern for a malignant primary cardiac tumor; however, imaging findings alone were not sufficient for definitive histologic diagnosis.

### Therapeutic intervention

The patient underwent thoracoscopic-assisted resection of the left ventricular mass and mitral valve repair under cardiopulmonary bypass. Intraoperatively, a gelatinous, multilobulated mass measuring approximately 5 cm × 5 cm was found to have a broad-based attachment near the left ventricular apex, consistent with the preoperative real-time TTE findings. Grossly complete resection was achieved intraoperatively, and the specimen was submitted for pathologic examination. Concomitant mitral annuloplasty was performed using a 28-mm prosthetic ring. The procedure was uneventful.

### Pathologic diagnosis

Histologic examination showed mildly atypical spindle cells resembling fibroblasts or myofibroblasts, predominantly arranged in fascicles, with focal perivascular growth. Mitotic figures were readily identified, and the stroma showed mild myxoid change. No tumor necrosis was identified. Immunohistochemistry showed positivity for vimentin, smooth muscle actin, MDM2, and p16, with partial positivity for CD34 and STAT6; calretinin, pancytokeratin, S100, and p53 were negative. The Ki-67 proliferation index was approximately 30% in hotspot areas. Fluorescence *in situ* hybridization demonstrated amplification of MDM2 and CDK4, with MDM2/CSP12 and CDK4/CSP12 ratios of 5.17 and 4.30, respectively. The integrated histomorphologic, immunophenotypic, and molecular findings supported the diagnosis of intimal sarcoma (Figure 3).

**Figure 3:**
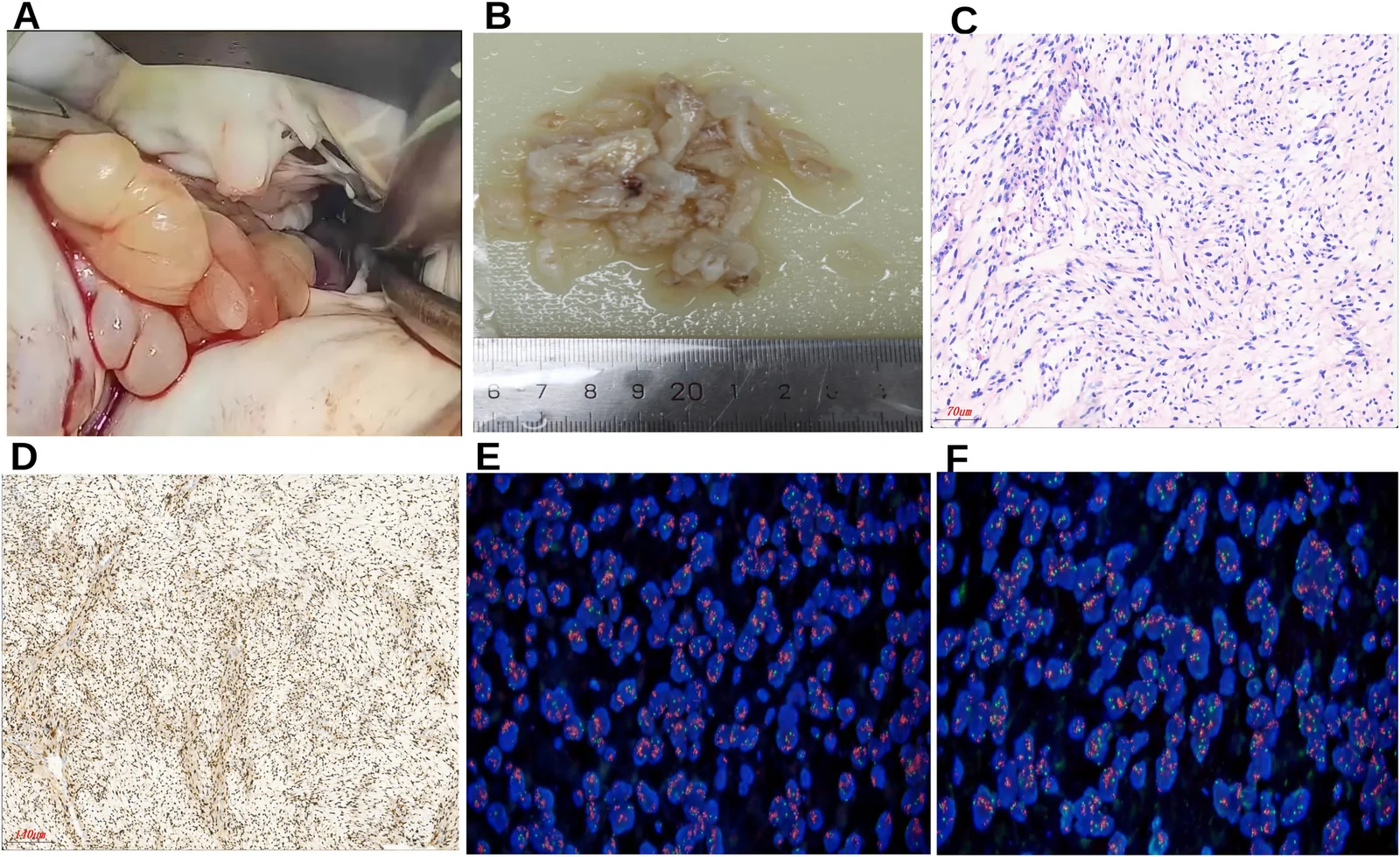
Operative, gross pathologic, histologic, immunohistochemical, and molecular pathologic findings. **(A)** Intraoperative photograph showing a large gelatinous, multilobulated mass within the left ventricle. **(B)** Gross specimen demonstrating the gray-white, soft, gelatinous, multilobulated appearance of the resected tumor. **(C)** Hematoxylin and eosin staining shows mildly atypical spindle cells resembling fibroblasts or myofibroblasts, predominantly arranged in fascicles, with focal perivascular growth, readily identified mitotic figures, and mild myxoid stromal change (×40; scale bar, 70 μm); tumor necrosis was not identified. **(D)** Immunohistochemical staining shows nuclear positivity for MDM2 in tumor cells (×20; scale bar, 140 μm). **(E)** Fluorescence *in situ* hybridization demonstrates MDM2 amplification, with an MDM2/CSP12 ratio of 5.17. **(F)** Fluorescence *in situ* hybridization demonstrates CDK4 amplification, with a CDK4/CSP12 ratio of 4.30.

### Follow-up and outcomes

Post-resection intraoperative TEE demonstrated no visible residual left ventricular mass and showed the result of the concomitant mitral valve repair (Supplementary Video S5). At approximately 2 weeks after surgery, follow-up TTE showed no residual or recurrent left ventricular mass. Left ventricular systolic function was preserved, with only minimal residual mitral regurgitation. The peak left ventricular outflow tract velocity was 0.92 m/s, with no evidence of residual outflow tract obstruction. 18F-FDG PET/CT performed at approximately the same time showed no definite extracardiac primary tumor, obvious distant metastasis, or abnormal hypermetabolic activity at the left ventricular surgical site suggestive of residual tumor. At multidisciplinary review, microscopic residual tumor at the surgical margin could not be excluded. Because of the aggressive behavior of cardiac intimal sarcoma and the possibility of residual disease, postoperative systemic therapy was recommended, with chemotherapy planned for approximately 3 weeks after surgery. Chest and upper abdominal CT after every two treatment cycles was recommended for surveillance. At the time of manuscript revision, information confirming whether systemic therapy had subsequently been started was not available. The patient remained clinically stable during this early postoperative period.

## Discussion

Primary cardiac intimal sarcoma is a rare malignant mesenchymal neoplasm that predominantly involves the left-sided cardiac chambers, while isolated left ventricular involvement is exceptionally uncommon. Clinical and imaging findings are nonspecific, and the lesion may be mistaken for myxoma, thrombus, or another cardiac mass. Reported left ventricular intimal sarcomas have presented as large intracavitary or infiltrative masses, sometimes with left ventricular outflow tract obstruction, but their echocardiographic appearances remain heterogeneous (4–7).

The most distinctive imaging feature in this case was the irregular multilobulated architecture with slit-like interlobular spaces. Real-time TTE demonstrated the relative motion of individual lobules and more clearly suggested a broad-based attachment near the apical interventricular septum. Low-velocity color Doppler demonstrated residual flow around the mass and within some clefts, explaining how intracavitary flow was partly maintained despite near-complete cavity occupation. Continuous-wave Doppler showed only mild outflow tract obstruction despite the large tumor burden. We speculate that the marked mobility of the mass and the interlobular flow channels helped preserve partial flow pathways.

TTE and intraoperative TEE provided complementary information in this patient. Visualization of a cardiac mass can vary with the acoustic window, imaging plane, and probe orientation (8, 9). The relatively apical attachment may have been less conspicuous on TEE because the apical left ventricular structures were in the far field of the transesophageal acoustic window. Anesthesia-related changes in ventricular loading and chamber geometry may also have brought the large mass closer to the ventricular wall and reduced the surrounding blood-tissue interface. These factors offer plausible explanations for the difference between the preoperative TTE and intraoperative TEE appearances, but the observation is specific to this case.

No single echocardiographic feature reliably establishes the histologic type of a cardiac mass. Papillary fibroelastomas may be highly mobile and frond-like but are generally small and most often arise from valvular surfaces (10). Myxomas are also mobile but usually arise from the left atrium with a more discrete attachment. These imaging features may help narrow the differential diagnosis, but substantial overlap exists among cardiac masses, and definitive distinction from benign tumors or thrombus requires pathologic and molecular evaluation. In this case, operative findings, histopathology, immunohistochemistry, and molecular testing were essential for diagnosis. MDM2 amplification, sometimes accompanied by CDK4 amplification, is an important diagnostic feature of intimal sarcoma (1–3).

The principal strength of this case lies in the multimodality characterization of a rare primary left ventricular intimal sarcoma, with correlation among imaging, operative findings, histopathology, immunohistochemistry, and molecular findings. Several limitations should be acknowledged. Cardiac MRI was not obtained before surgery because the clinical team prioritized prompt surgical intervention for the large, highly mobile left ventricular mass with potential hemodynamic consequences. The absence of preoperative CMR limited additional tissue characterization and independent assessment of possible myocardial involvement. Real-time 3D TEE was not performed, resulting in asymmetry between the transthoracic and transesophageal imaging protocols and limiting direct comparison between the two examinations. The postoperative follow-up reported here remains early. Although postoperative PET/CT showed no definite extracardiac primary tumor or obvious distant metastatic lesions, longer-term surveillance is required because of the risk of local recurrence and systemic metastasis. Multidisciplinary evaluation recommended postoperative systemic therapy and serial chest and upper abdominal CT after every two treatment cycles. Longer-term treatment response and oncologic outcomes were not available at the time of manuscript revision.

## Conclusion

This case describes a giant primary left ventricular intimal sarcoma with complex multilobulated morphology and associated hemodynamic effects. TTE, TEE, and cardiac CTA contributed complementary anatomic and hemodynamic information, whereas the final diagnosis depended on operative findings together with histopathologic, immunohistochemical, and molecular evaluation. The different appearances on preoperative TTE and intraoperative TEE in this patient likely reflected a combination of imaging-related and physiologic factors.

## Data Availability

The original contributions presented in the study are included in the article/Supplementary Material, further inquiries can be directed to the corresponding author.

## Supplementary material

Supplementary Video S1 Conventional two-dimensional grayscale TTE demonstrating the overall extent of the left ventricular mass and its marked mobility throughout the cardiac cycle.

Supplementary Video S2 Real-time two-dimensional pseudocolor TTE demonstrating the irregular multilobulated configuration, movement of individual lobules, slit-like interlobular spaces, and probable broad-based attachment near the apical interventricular septum.

Supplementary Video S3 Low-velocity-scale color Doppler TTE demonstrating residual low-velocity blood flow within some interlobular clefts of the mass.

Supplementary Video S4 Pre-resection intraoperative biplane TEE after induction of general anesthesia showed the mass nearly occupying the entire left ventricular cavity. Compared with preoperative TTE, the attachment site and detailed multilobulated architecture were less clearly visualized. Real-time three-dimensional TEE was not performed in this case.

Supplementary Video S5 Post-resection intraoperative TEE demonstrating no visible residual left ventricular mass and the result of concomitant mitral valve repair
